# Gemcitabine–cisplatin versus MVAC chemotherapy for urothelial carcinoma: a nationwide cohort study

**DOI:** 10.1038/s41598-023-30356-x

**Published:** 2023-03-06

**Authors:** Yong Seong Lee, Moon Soo Ha, Jong Hyun Tae, In Ho Chang, Tae-Hyoung Kim, Soon Chul Myung, Tuan Thanh Nguyen, Myoungsuk Kim, Kyung-Eun Lee, Yuwon Kim, Hyun-ki Woo, Dae-Sung Kyoung, Hasung Kim, Se Young Choi

**Affiliations:** 1 Department of Urology, Chung-Ang University Gwangmyeong Hospital, Chung-Ang University College of Medicine, Gyeonggi-Do, Republic of Korea; 2 Department of Urology, Hyundae General Hospital, Chung-Ang University College of Medicine, Gyeonggi-Do, Republic of Korea; 3 Department of Urology, Chung-Ang University Hospital, Chung-Ang University College of Medicine, 102, Heukseok-Ro, Dongjak-Gu, Seoul, 06973 Republic of Korea; 4 Department of Urology, Cho Ray Hospital, University of Medicine and Pharmacy at Ho Chi Minh City, Ho Chi Minh City, Vietnam; 5 Data Science Team, Evidnet. Co., Ltd, Seoul, Republic of Korea; 6 Data Science Team, Hanmi Pharm. Co., Ltd, Seoul, Republic of Korea

**Keywords:** Diseases, Medical research, Oncology, Urology

## Abstract

This study assessed the trends in methotrexate, vinblastine, doxorubicin, and cisplatin (MVAC) and gemcitabine–cisplatin (GC) regimens in Korean patients with metastatic urothelial carcinoma (UC) and compared the side effects and overall survival (OS) rates of the two regimens using nationwide population-based data. The data of patients diagnosed with UC between 2004 and 2016 were collected using the National Health Insurance Service database. The overall treatment trends were assessed according to the chemotherapy regimens. The MVAC and GC groups were matched by propensity scores. Cox proportional hazard analysis and Kaplan–Meier analysis were performed to assess survival. Of 3108 patients with UC, 2,880 patients were treated with GC and 228 (7.3%) were treated with MVAC. The transfusion rate and volume were similar in both the groups, but the granulocyte colony-stimulating factor (G-CSF) usage rate and number were higher in the MVAC group than in the GC group. Both groups had similar OS. Multivariate analysis revealed that the chemotherapy regimen was not a significant factor for OS. Subgroup analysis revealed that a period of ≥ 3 months from diagnosis to systemic therapy enhanced the prognostic effects of the GC regimen. The GC regimen was widely used as the first-line chemotherapy in more than 90% of our study population with metastatic UC. The MVAC regimen showed similar OS to the GC regimen but needed greater use of G-CSF. The GC regimen could be a suitable treatment option for metastatic UC after ≥ 3 months from diagnosis.

## Introduction

Urothelial carcinoma (UC) occurs on the mucosal surface of the renal pelvis, ureter, bladder, and urethra. Bladder cancer (BCa) is the most common UC, being the 10th most prevalent cancer worldwide^1^. However, upper tract UC (UTUC) accounts for less than 10% of UCs^2^. In patients with muscle-invasive BCa that cannot be completely surgically resected or is accompanied by metastasis, the prognosis is so poor that the long-term disease-free survival rate is reported to be less than 5%^3^. Methotrexate, vinblastine, doxorubicin, and cisplatin (MVAC) and gemcitabine–cisplatin (GC) are the standard chemotherapy regimens for treating locally advanced or metastatic disease. In the 1980s, Stenberg et al. assessed 133 patients with UC treated with MVAC and reported complete remission (CR) in 36% of patients, although additional surgical resection of the remnant tumor^4^. Patients in CR survived for a median of 38 months; however, at least 68% of patients in CR experienced disease recurrence^4^. In a randomized controlled trial (RCT) of advanced UC, MVAC was superior to single-agent cisplatin in terms of the response rate, progression-free survival (PFS), and overall survival (OS)^5^. However, MVAC showed significant toxicity in the form of drug-related mortality (3%) and myelosuppression (58%)^4^.

In the 2000s, an RCT compared the survival advantage and safety of MVAC and GC regimens^6^. Although the OS and response rate were similar in both the regimens, the MVAC regimen was associated with a higher rate of grade 3/4 anemia, thrombocytopenia, and neutropenia^6^. The GC regimen has been widely used as the standard first-line chemotherapy because of its lower toxicity. To reduce the side effects of the established MVAC regimen, the efficacy of a high-dose MVAC regimen with approximately twice the intensity of cisplatin and granulocyte colony-stimulating factor (G-CSF) was assessed in an RCT^7^. High-dose MVAC led to a superior CR rate (21%) than MVAC, with the median OS being similar in both the regimens^7^. Moreover, high-dose MVAC resulted in a lower incidence of neutropenia and neutropenic fever^7^.

There is no report on UC treatment trends in Korea. Therefore, we assessed the trends in the MVAC and GC regimens in Korean patients with UC and compared the side effects and OS rates of the two regimens using nationwide population-based data.

## Materials and methods

### Database

Data were collected using the National Health Insurance Service (NHIS) database. The NHIS is a universal health coverage system in South Korea. Over 97% of the Korean population (over 50 million individuals) is enrolled in the NHIS. NHIS provided anonymized data only to authorized persons in limited space after the approvement of institutional review board. NHIS database did not include tumor characteristics such as stage and grade.

### Study design

**Figure 1:**
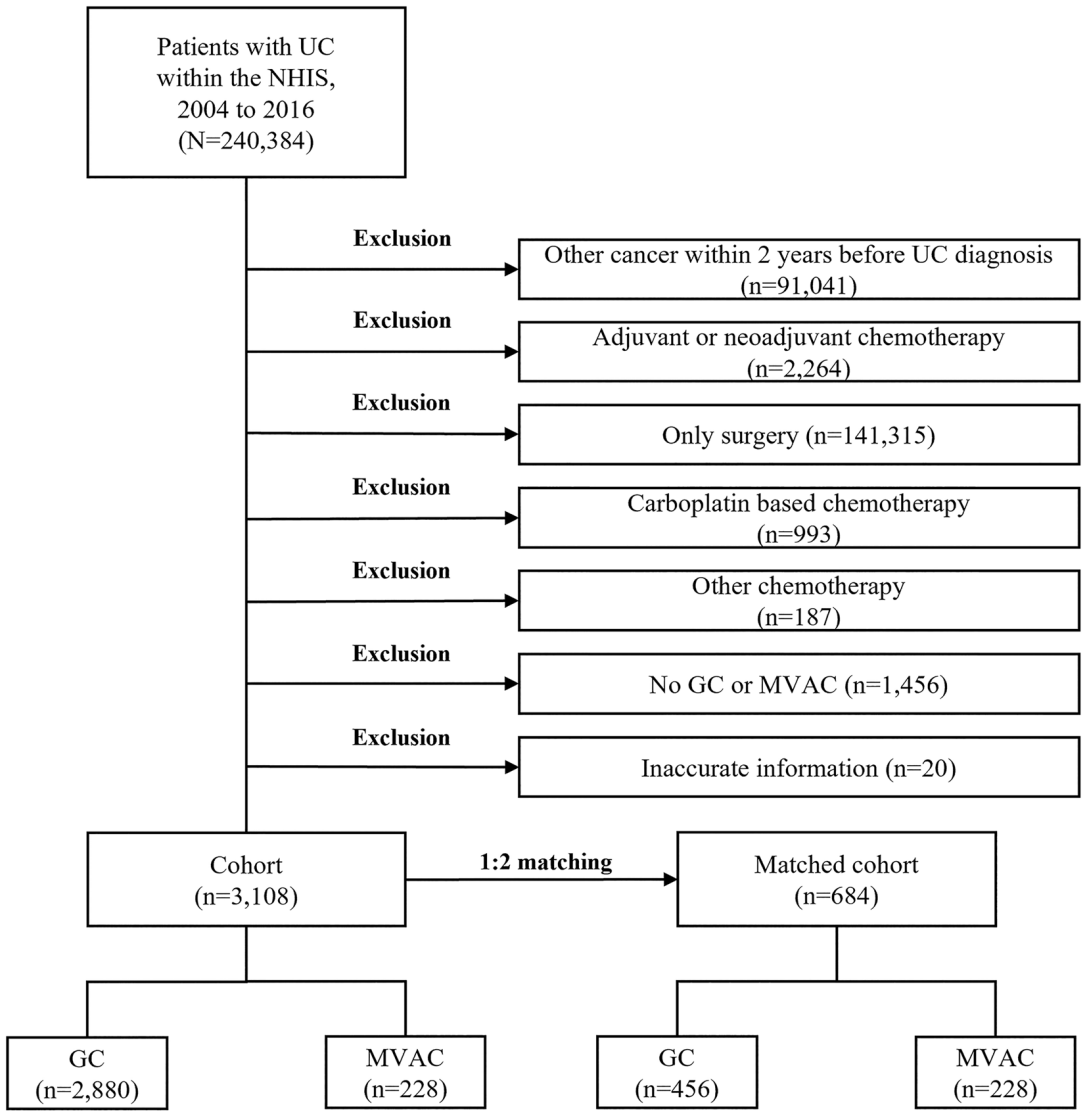
Flow chart of the study design. UTUC, upper tract urothelial carcinoma; NHIS, National Health Insurance Service; AC, adjuvant chemotherapy; NAC, neoadjuvant chemotherapy; NUx, nephroureterectomy.

The study period for the original cohort was between 2002 and 2018; we chose a 2-year wash-out period (2002 to 2003) and a 2-year follow-up period (2017 to 2018). All the patients had UC (International Classification of Diseases [ICD] diagnostic codes C65 [ureter], C66 [pelvis], and C67 [bladder]) between 2004 and 2016. All cases were confirmed by pathology or cytology with metastatic radiology. Inclusion criteria was the usage of GC or MVAC chemotherapy with UC diagnostic codes. Patients who were diagnosed as having other cancers within 2 years, received adjuvant or neoadjuvant chemotherapy, underwent only surgery, received other chemotherapy, and had inaccurate data were excluded. Neoadjuvant chemotherapy was defined as chemotherapy administered during 3 months before nephroureterectomy (NUx) or radical cystectomy (RCx), and adjuvant chemotherapy was defined as chemotherapy administered during 3 months after NUx or RCx. A flow chart of the study design is displayed in Fig. 1.

### Variables

The study variables included age, sex, diagnosis, year of diagnosis, and Charlson Comorbidity Index (CCI) score. The comorbidity status was calculated using the CCI^8^. Variables that have a strong influence on the patient’s prognosis were used in propensity score matching (PSM). Moreover, data regarding surgeries, G-CSF use, hospitalization, transfusion, radiation, and survival were collected. We used the average exchange rate between the Korean won and the United States dollar from 2002 to 2018 (KRW 1120 = USD 1).

### Statistical analyses

Clinical trends are expressed as means ± standard deviations or numbers with percentages. Differences between the groups were compared using Student’s t-test for continuous variables and the chi-squared test for categorical variables. OS was calculated from the date of diagnosis to the date of the last follow-up or death using the Kaplan–Meier method with the log-rank test. PSM was performed based on age, sex, diagnosis, year of diagnosis, and CCI score. A 1:2 nearest matching was performed. The Cox proportional hazards model was used for multivariate analysis. All statistical analyses were performed using SAS v.7.0 (SAS Institute Inc., Cary, NC, USA) and R software, version 4.0.1 (R Foundation for Statistical Computing, Vienna, Austria). A *p* value < 0.05 was considered statistically significant.

## Results

The total cohort consisted of 3,108 patients. Of these, 2880 patients were treated with GC (GC group) and 228 were treated with MVAC (MVAC group) (Table 1). The mean patient age was higher in the GC group (66.4 ± 10.6 years) than in the MVAC group (63.3 ± 12.6 years, *p* < 0.001). The number of male patients was higher in the MVAC group than in the GC group (85.1% vs. 77.9%, *p* = 0.014). The use of GC was the highest in 2013–2016 (37.5%), whereas that of MVAC was the highest in 2004–2008 (39.5%). After PSM, all the differences were balanced by the variables with a standardized mean difference < 0.1.

**Table 1:** Baseline Characteristics of the Study Population.

	Before match			After match			
	GC (n = 2880)	MVAC (n = 228)	*p*-value	GC (n = 456)	MVAC (n = 228)	*p*-value	SMD
*Diagnosis*			0.940			0.605	0.051
BCa	2451 (85.1%)	195 (85.5%)		398 (87.3%)	195 (85.5%)		
UTUC	429 (14.9%)	33 (14.5%)		58 (12.7%)	33 (14.5%)		
Age	66.4 ± 10.6	63.3 ± 12.6	< 0.001	63.7 ± 10.8	63.3 ± 12.6	0.627	< 0.001
	68.0 [60.0;74.0]	65.5 [56.5;72.0]		66.0 [57.0;71.0]	65.5 [56.5;72.0]		
*Age group*			< 0.001			1.000	< 0.001
< 55	399 (13.9%)	47 (20.6%)		94 (20.6%)	47 (20.6%)		
55–64	680 (23.6%)	58 (25.4%)		116 (25.4%)	58 (25.4%)		
65–74	1123 (39.0%)	91 (39.9%)		182 (39.9%)	91 (39.9%)		
≥ 75	678 (23.5%)	32 (14.0%)		64 (14.0%)	32 (14.0%)		
*Sex*			0.014			1.000	< 0.001
Male	2243 (77.9%)	194 (85.1%)		388 (85.1%)	194 (85.1%)		
Female	637 (22.1%)	34 (14.9%)		68 (14.9%)	34 (14.9%)		
*Diagnosis year*			0.018			1.000	< 0.001
2004–2008	887 (30.8%)	90 (39.5%)		180 (39.5%)	90 (39.5%)		
2009–2012	914 (31.7%)	58 (25.4%)		116 (25.4%)	58 (25.4%)		
2013–2016	1079 (37.5%)	80 (35.1%)		160 (35.1%)	80 (35.1%)		
*CCI*			0.147			1.000	< 0.001
0,1	1075 (37.3%)	100 (43.9%)		200 (43.9%)	100 (43.9%)		
2,3	1102 (38.3%)	78 (34.2%)		156 (34.2%)	78 (34.2%)		
≥ 4	703 (24.4%)	50 (21.9%)		100 (21.9%)	50 (21.9%)		

Data are presented as n (%), mean ± standard deviation, or median [interquartile range].

GC, Gemcitabine + Cisplatin Chemotherapy; MVAC, Methotrexate + Vinblastine + Doxorubicin + Cisplatin Chemotherapy;SMD, Standarized Mean Difference; BCa, Bladder cancer; UTUC, Upper tract urothelial carcinoma; CCI, Charlson Comorbidity Index.

**Figure 2:**
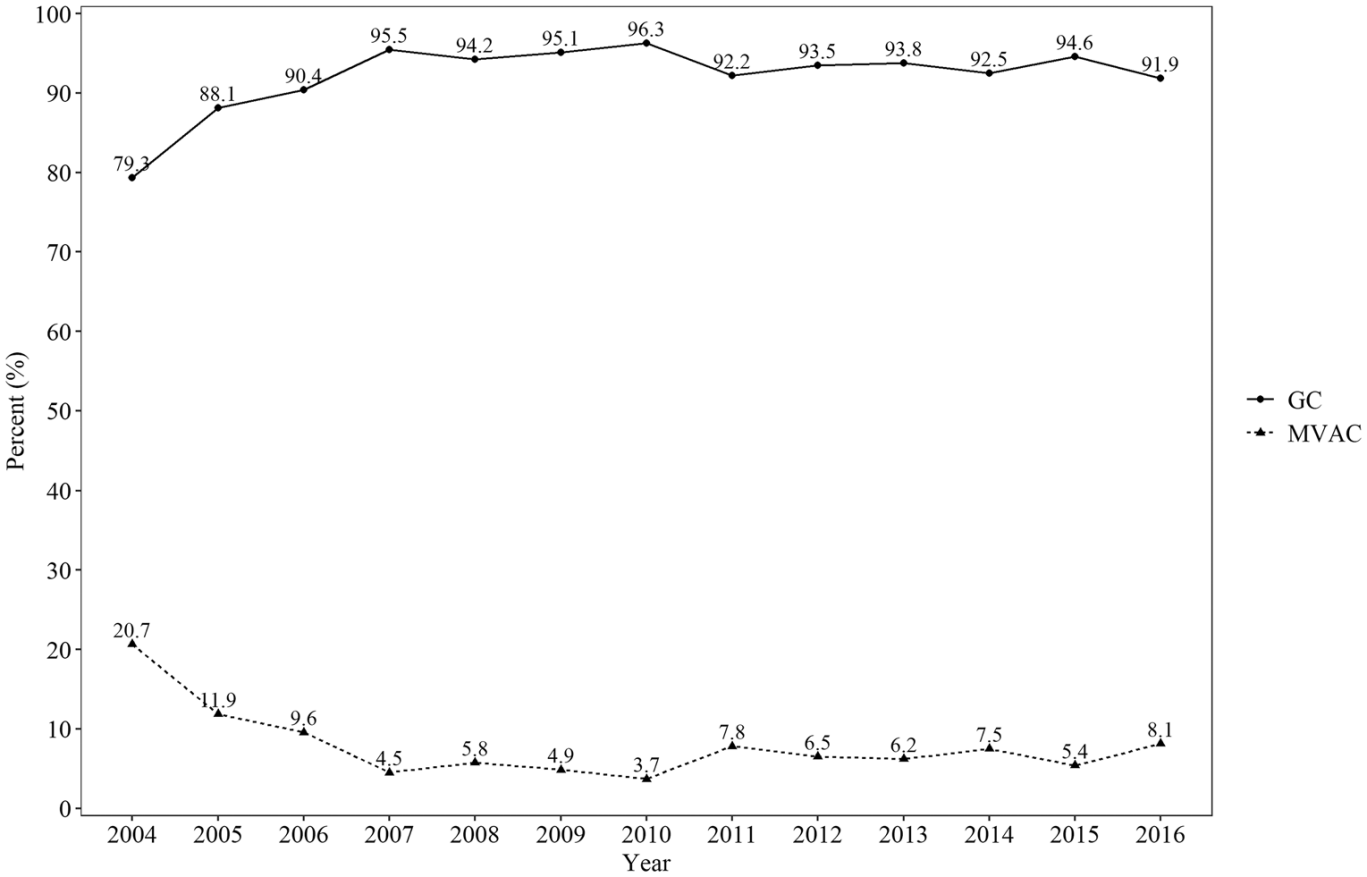
Upper tract urothelial carcinoma treatment regimens.

The chemotherapy regimens utilized for UC are presented in Fig. 2. MVAC was used for 7.3% of the total study population. The usage rate of MVAC was 20.7% in 2004 and 8.1% in 2016.

The clinical trends in the chemotherapy regimens are presented in Table 2. The period from diagnosis to chemotherapy was a median of 3 months in the GC group and 2.9 months in the MVAC group (*p* = 0.624). The period from radical surgery to chemotherapy was a median of 10.3 months in the GC group and 10.4 months in the MVAC group (*p* = 0.894). The percentage of patients undergoing radical surgery was higher in the GC group than in the MVAC group (16.9% vs. 10.5%, *p* = 0.036). The transurethral resection of bladder tumor (TUR-BT) data regarding the chemotherapy utilization rate, number, and period were similar between the groups. The duration of hospitalization, except for that for chemotherapy, was also similar between the groups (*p* = 0.438). Moreover, the admission rates during the total period and within 3 months were similar between the groups. Although the transfusion rate and volume were similar between the groups, the G-CSF usage rate and number were higher in the MVAC group than in the GC group (*p* < 0.001 and *p* = 0.034, respectively). The G-CSF usage rate within 3 months was also higher in the MVAC group than in the GC group (*p* < 0.001). The radiotherapy utilization rate and 180-day mortality rate did not differ between the groups. The costs of 1 year and total period were lower in the MVAC group than in the GC group (*p* = 0.002 and *p* = 0.019, respectively).

**Table 2:** Clinical trends after the treatments.

	Before match			After match		
	GC (n = 2880)	MVAC (n = 228)	*p*-value	GC (n = 456)	MVAC (n = 228)	*p*-value
Period from diagnosis to ST (months)	12.2 ± 19.5	10.5 ± 17.8	0.216	11.2 ± 17.8	10.5 ± 17.8	0.624
	3.5 [1.1;14.6]	2.9 [1.0;11.3]		3.0 [1.0;14.0]	2.9 [1.0;11.3]	
Period from NUx (or RCx) to ST (months)	18.3 ± 18.6	17.3 ± 20.0	0.762	16.8 ± 16.0	17.3 ± 20.0	0.894
	12.1 [5.6;24.1]	10.4 [5.2;18.9]		10.3 [5.7;22.0]	10.4 [5.2;18.9]	
Previous NUx (or RCx) (%)	445 (15.5%)	24 (10.5%)	0.057	77 (16.9%)	24 (10.5%)	0.036
Previous TUR-BT (%)	2061 (71.6%)	159 (69.7%)	0.609	327 (71.7%)	159 (69.7%)	0.655
Number of previous TUR-BT	1.8 ± 1.4	1.7 ± 1.5	0.598	1.7 ± 1.3	1.7 ± 1.5	0.986
	1.0 [1.0; 2.0]	1.0 [1.0; 2.0]		1.0 [1.0; 2.0]	1.0 [1.0; 2.0]	
Period from last TUR-BT to ST (months)	13.2 ± 20.2	12.0 ± 16.9	0.387	13.9 ± 21.5	12.0 ± 16.9	0.272
	4.9 [1.0;16.6]	3.9 [1.0;17.4]		4.9 [1.0;16.6]	3.9 [1.0;17.4]	
Number of TUR-BT after ST	1.9 ± 1.5	1.9 ± 1.4	0.937	2.1 ± 1.6	1.9 ± 1.4	0.581
	1.0 [1.0; 2.0]	1.0 [1.0; 2.5]		1.0 [1.0; 2.0]	1.0 [1.0; 2.5]	
Hospitalization duration except ST (days)	58.6 ± 89.6	65.1 ± 162.0	0.593	55.1 ± 95.1	65.1 ± 162.0	0.438
	37.0 [16.0;71.0]	36.5 [18.0;76.5]		37.0 [16.0;71.0]	36.5 [18.0;76.5]	
Admission rate except ST (%)	2505 (87.0%)	184 (80.7%)	0.010	394 (86.4%)	184 (80.7%)	0.067
Admission rate except ST within 3-months (%)	652 (22.6%)	57 (25.0%)	0.462	96 (21.1%)	57 (25.0%)	0.284
Transfusion after ST (%)	1842 (64.0%)	133 (58.3%)	0.104	281 (61.6%)	133 (58.3%)	0.455
Volume of transfusion (packs)	8.8 ± 9.8	9.1 ± 9.0	0.764	8.7 ± 10.3	9.1 ± 9.0	0.764
	6.0 [2.0;11.0]	6.0 [4.0;11.0]		5.0 [2.0;10.0]	6.0 [4.0;11.0]	
G-CSF usage rate (%)	1007 (35.0%)	127 (55.7%)	< 0.001	157 (34.4%)	127 (55.7%)	< 0.001
The number of G-CSF usage (N)	2.5 ± 2.7	2.9 ± 3.0	0.190	2.2 ± 1.9	2.9 ± 3.0	0.034
	2.0 [1.0; 3.0]	2.0 [1.0; 3.5]		1.0 [1.0; 3.0]	2.0 [1.0; 3.5]	
The rate of G-CSF usage within 3-months (%)	451 (15.7%)	82 (36.0%)	< 0.001	71 (15.6%)	82 (36.0%)	< 0.001
The rate of radiotherapy	372 (12.9%)	32 (14.0%)	0.703	65 (14.3%)	32 (14.0%)	1.000
180-day mortality during primary ST	818 (28.4%)	80 (35.1%)	0.039	131 (28.7%)	80 (35.1%)	0.107
Costs of 1 year after the index date (1000 USD)	14.6 ± 10.8	13.1 ± 11.5	< 0.00	14.5 ± 11.4	13.1 ± 11.5	0.002
	12.3 [7.9;18.0]	9.3 [5.1; 18.9]	1	12.1 [7.8; 17.4]	9.3 [5.1; 18.9]	
Costs of total period (1000 USD)	25.6 ± 20.4	24.2 ± 22.3	0.006	25.8 ± 19.3	24.2 ± 22.3	0.019
	20.5 [12.9; 32.4]	18.5 [9.4; 31.9]		20.9 [12.7; 32.9]	18.5 [9.4; 31.9]	

Data are presented as n (%), mean ± standard deviation, or median [interquartile range].

GC, Gemcitabine + Cisplatin Chemotherapy; MVAC, Methotrexate + Vinblastine + Doxorubicin + Cisplatin Chemotherapy; ST, systemic therapy; NUx, Nephroureterectomy; RCx, Radical cystectomy; TUR-BT, Transurethral resection of bladder tumor; G-CSF, Granulocyte colony-stimulating factor; UDS, United States Dollar.

**Figure 3:**
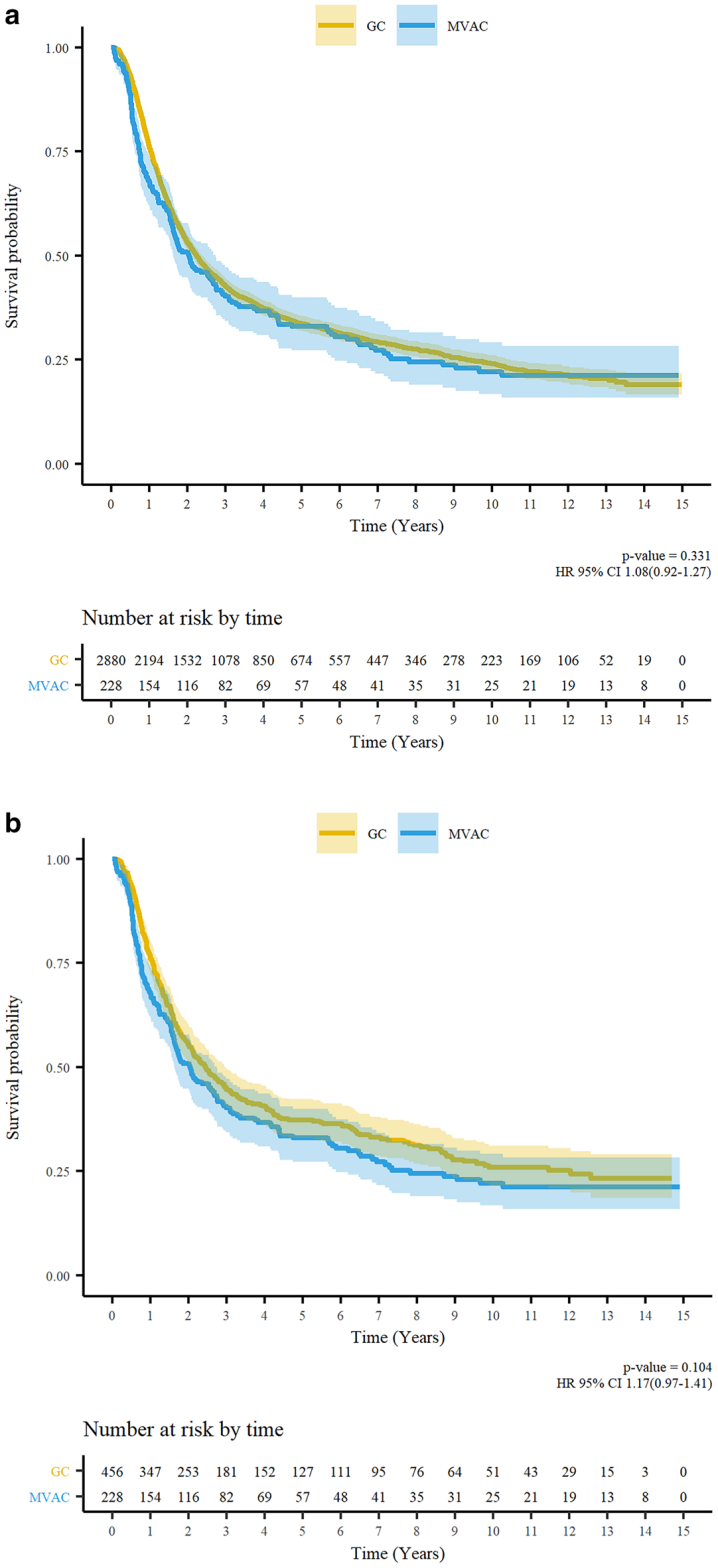
Kaplan–Meier survival curves and log-rank tests for overall survival (**A**) before propensity score matching and (**B**) after propensity score matching. AC, adjuvant chemotherapy; NAC, neoadjuvant chemotherapy; HR, hazard ratio; CI, confidence interval.

**Figure 4:**
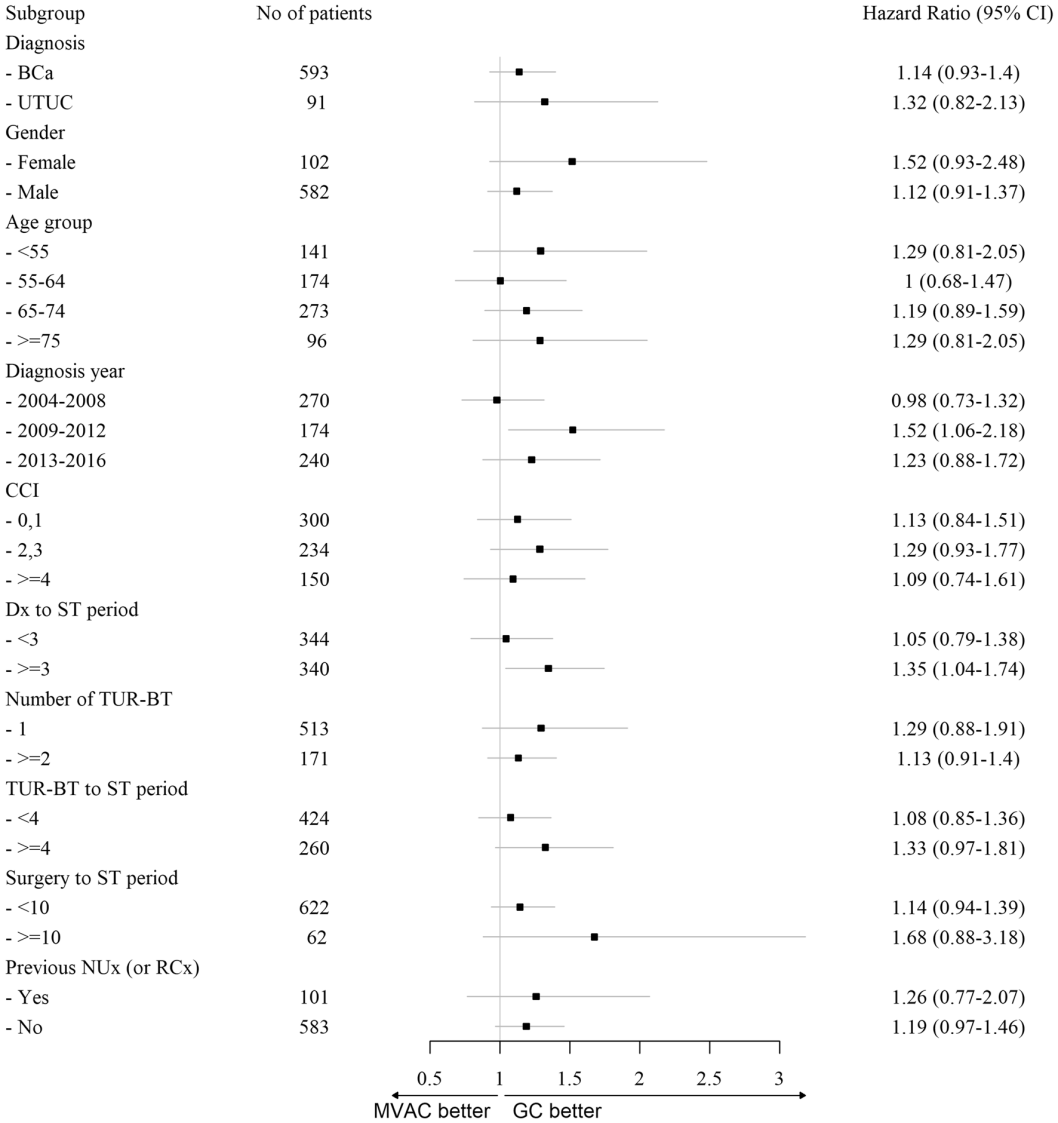
Forest plot classified by chemotherapy regimen. BCa, bladder cancer; UTUC, upper tract urothelial carcinoma; CCI, Charlson Comorbidity Index; Dx, Diagnosis; ST, systemic therapy; TUR-BT, transurethral resection of bladder tumor.

Before PSM, the 5-year OS rates were 23.4% in the GC group and 25.0% in the MVAC group. Based on the Kaplan–Meier curve, the OS was similar in both the groups (*p* = 0.331, Fig. 3A). The median OS was 39.6 months in the GC group and 37 months in the MVAC group. After PSM, the 5-year OS rate was 27.9% in the GC group and 25.0% in the MVAC group. The MVAC group also showed similar OS to the GC group based on the Kaplan–Meier curve (*p* = 0.104, Fig. 3B). Multivariate analysis (Table 3) revealed that the chemotherapy regimen (GC or MVAC) was not a significant factor for OS before PSM (hazard ratio [HR] 1.16, 95% confidence interval [CI] 0.99–1.36, *p* = 0.068) and after PSM (HR 1.19, 95% CI 0.98–1.43, *p* = 0.078). Patients aged 65–74 years (HR 1.58, 95% CI 1.22–2.07, *p* = 0.001) and ≥ 75 years (HR 1.93, 95% CI 1.38–2.70, *p* < 0.001) had worse OS than those aged < 55 years. Moreover, patients with UTUC had worse OS than those with BCa (HR 1.59, 95% CI 1.22–2.05, *p* < 0.001). Subgroup analysis revealed that a period of ≥ 3 months from diagnosis to systemic therapy (HR 1.35, 95% CI 1.04–1.74) enhanced the prognostic effects of the GC regimen (Fig. 4).

**Table 3:** Hazard ratio and 95% CI of overall survival according to treatment.

	Before match				After match			
	Univariate	*p*-value	Multivariate	*p*-value	Univariate	*p*-value	Multivariate	*p*-value
*Treatment*								
GC	1		1		1		1	
MVAC	1.08(0.92–1.27)	0.331	1.16(0.99–1.36)	0.068	1.17(0.97–1.41)	0.104	1.19(0.98–1.43)	0.078
*Age*								
< 55	1		1		1		1	
55–64	1.2(1.03–1.4)	0.017	1.16(1–1.35)	0.055	1.27(0.95–1.69)	0.105	1.15(0.86–1.55)	0.348
65–74	1.49(1.29–1.71)	< 0.001	1.43(1.24–1.65)	< 0.001	1.64(1.26–2.13)	< 0.001	1.58(1.2–2.07)	0.001
≥ 75	1.86(1.6–2.16)	< 0.001	1.8(1.54–2.09)	< 0.001	2(1.46–2.73)	< 0.001	1.93(1.38–2.70)	< 0.001
*Sex*								
Male	1		1		1		1	
Female	0.95(0.86–1.06)	0.374	1.05(0.94–1.16)	0.409	1(0.77–1.29)	0.973	1.04(0.8–1.37)	0.757
*CCI*								
0,1	1		1		1		1	
2,3	1.15(1.04–1.27)	0.005	1.08(0.97–1.19)	0.150	1.16(0.94–1.42)	0.167	1.1(0.89–1.37)	0.376
≥ 4	1.39(1.25–1.56)	< 0.001	1.23(1.09–1.38)	0.001	1.43(1.13–1.8)	0.003	1.18(0.91–1.52)	0.216
*Diagnosis year*								
2004–2008	1		1		1		1	
2009–2012	1.15(1.04–1.28)	0.007	1.08(0.97–1.2)	0.162	1.22(0.98–1.53)	0.080	1.14(0.9–1.44)	0.290
2013–2016	1.07(0.96–1.19)	0.209	0.98(0.88–1.1)	0.758	1.29(1.04–1.61)	0.023	1.2(0.96–1.52)	0.112
*Diagnosis*								
BCa	1		1		1		1	
UTUC	1.27(1.13–1.42)	< 0.001	1.3(1.16–1.46)	< 0.001	1.45(1.13–1.87)	0.004	1.59(1.22–2.05)	< 0.001

GC, Gemcitabine + Cisplatin Chemotherapy; MVAC, Methotrexate + Vinblastine + Doxorubicin + Cisplatin Chemotherapy; CCI, Charlson Comorbidity Index; BCa, Bladder cancer; UTUC, Upper tract urothelial carcinoma.

## Discussion

Of the patients with UC who received the GC or MVAC regimen as the first-line chemotherapy, 462 patients had UTUC (14.9%) and 2,646 patients had BCa (85.1%). The MVAC regimen was used for 228 patients (7.8%), and the GC regimen was used for 2880 patients (92.2%). The MVAC regimen was more commonly used for younger and male patients than the GC regimen; however, after PSM, the differences were well balanced. The G-CSF usage rate and number were higher in the MVAC group. However, the admission rate, hospitalization duration, transfusion rate, and 180-day mortality rate did not differ between the two groups. Moreover, the median OS was similar between the two groups. The GC group showed better OS than the MVAC group for cases with ≥ 3 months from diagnosis to chemotherapy.

UCs include UTUC and BCa, which have histological similarities and are associated with the urinary system. Because of these similarities and the relative rarity of UTUC, the standard chemotherapy for UTUC has been chosen to be the same as that for BCa. However, there has been a debate on combining both UTUC and BCa as a single disease entity. While less than a quarter of patients with BCa had muscle invasiveness, more than half of patients with UTUC were found to be in the advanced stage at diagnosis in a previous study^9^. This could be attributed to the thin muscle layer of the upper urinary tract. Based on international consensus, the molecular subtypes of muscle-invasive BCa are as follows: luminal papillary (24%), luminal nonspecified (8%), luminal unstable (15%), stroma-rich (15%), basal/squamous (35%), and neuroendocrine-like (3%) subtypes^10^. This transcriptomic molecular classification can help predict the prognosis or drug response^10,11^. The genomic characteristics of UTUC are lesser known than those of BCa. Audenet et al. reported that the mutation burden of UTUC was significantly higher than that of BCa and there were differences in the prevalence of genomic alterations between the two cancers^12^. The molecular subtypes showed different sensitivities to chemotherapy^13^. In our study, UTUC showed a poorer prognosis than BCa among patients treated with first-line chemotherapy.

Cisplatin is platinum-based chemotherapeutic agent that binds to DNA and inhibits DNA replication; it is commonly used in both regimens^14^. While gemcitabine is used in the GC regimen, methotrexate, vinblastine, and doxorubicin are used in the MVAC regimen. Gemcitabine inhibits DNA synthesis as a nucleoside analog and results in cell death^15^. Methotrexate also inhibits DNA synthesis through an antifolate mechanism^16^. Doxorubicin inhibits topoisomerase II, thereby preventing DNA replication^17^. Gemcitabine, methotrexate, and doxorubicin are classified as antimetabolites. Vinblastine disrupts the microtubule as a mitotic inhibitor^18^. Although its mechanism of action is mostly similar to that of DNA damage, the oncological outcomes and adverse effects can differ^19^ because they depend on the target DNA lesion, cell differentiation, and drug exposure duration^19^. Methotrexate and doxorubicin can cause bone marrow suppression, whereas gemcitabine can frequently cause hepatitis^19^. In our study, the G-CSF usage rate and number were higher in the MVAC group than in the GC group.

In Korea, the GC regimen is widely used as the first-line chemotherapy in more than 90% of patients with advanced UC. The use of the GC regimen may be preferred because of its tolerable toxicity and simplicity. A few studies have compared the efficacy and side effects of the MVAC and GC regimens in Korean patients and reported the GC regimen to be more effective and have lower side effects than the MVAC regimen^20^.

Our subgroup analysis revealed that the GC regimen was more effective than the MVAC regimen in patients who were treated after ≥ 3 months from the diagnosis of UC. The patients may experience recurrence after the primary surgery or delays resulting from complications of previous procedures or external causes. The coronavirus disease 2019 (COVID-19) pandemic can be one of the external causes, and many studies have reported that delays in the treatment of BCa can be harmful to patients^21,22^. Timely and accurate treatment is important for patients with UC; however, the GC regimen would be better than the MVAC regimen in treating UC after ≥ 3 months from diagnosis.

This study has some limitations. First, the small sample size of the MVAC group is asymmetric. We collected nearly complete enumeration data on chemotherapy from South Korea because insurance services cover the high cost of chemotherapy and most of the Korean population is enrolled in the NHIS. To the best of our knowledge, no study has reported the nationwide trends in GC and MVAC and compared their efficacy and safety in Korea. Second, the regimen was chosen by the clinicians; this may have led to a selection bias. Because perioperative chemotherapy cases and carboplatin cases were excluded, caution is required in interpreting the data based on actual clinical practice. To reduce the bias, we used PSM and found that the differences between GC and MVAC were well matched. Third, the NHIS data do not contain any tumor characteristics, such as pathological or radiological images. However, the insurance covers patients with UC who only prove metastasis by pathology or cytology with radiological images. Therefore, we assumed patients with metastatic UC who should use GC or MVAC chemotherapy in the NHIS using the operational definition, with the exclusion of perioperative chemotherapy. In the retrospective study, the overall survival between the GC and MVAC groups were adjusted for their backgrounds using statistical methods such as PSM and multivariate analysis. Finally, a high-dose MVAC regimen was not included in our study cohort because the NHIS did not cover this regimen during our study period. The NHIS recently added the high-dose MVAC regimen to its insurance coverage list. . Although we could not confirm the regimen of high-dose MVAC due to limitations of NHIS database, high-dose MVAC is the one of important first-line chemotherapies.

## Conclusions

In this nationwide cohort from Korea, the GC regimen was widely used as the first-line chemotherapy in more than 90% of patients with metastatic UC. The MVAC regimen resulted in similar OS to the GC regimen but needed greater use of G-CSF. Among UCs, metastatic UTUC has a poorer prognosis than metastatic BCa. The GC regimen could be a proper treatment option for metastatic UC after ≥ 3 months from diagnosis. These results could guide the treatment of patients in the presence of external delays, such as those caused by the COVID-19 pandemic.

## Data Availability

The data that support the findings of this study are available from the National Health Insurance Service but restrictions apply to the availability of these data, which were used under license for the current study, and so are not publicly available. Data are however available from the authors upon reasonable request and with permission of the National Health Insurance Service. Original raw data can be assessed through direct contact with the author, Se Young Choi (alse3025@gmail.com).
